# An Observational Study to Evaluate the Effect of L-ornithine L-aspartate in Patients With Overt Hepatic Encephalopathy Receiving Lactulose and Rifaximin

**DOI:** 10.7759/cureus.89949

**Published:** 2025-08-12

**Authors:** Saheli Das, Chinthaparthi Reddy, Sukanya Bhrugumalla, Radhika Soanker

**Affiliations:** 1 Clinical Pharmacology and Therapeutics, Nizam's Institute of Medical Sciences, Hyderabad, IND; 2 Gastroenterology, Nizam's Institute of Medical Sciences, Hyderabad, IND; 3 Pharmacology, All India Institute of Medical Sciences, Bibinagar, Hyderabad, IND

**Keywords:** hepatic encephalopathy, lactulose, lola, rifaximin, west haven criteria

## Abstract

Background: Hepatic encephalopathy (HE) is one of the most frequent complications and debilitating manifestations of liver disease. The condition ranges from a change in the cognitive status to finally progressing towards confusion, personality alterations, disorientation, lethargy, and coma. Lactulose, rifaximin, oral branched-chain amino acids (BCAA), intravenous L-ornithine L-aspartate (LOLA), and probiotics are used for the treatment.

Method: This was a prospective, observational, add-on, parallel-group study with a 12-month duration and a sample size of 53. Patients were divided into two study groups - one group received LOLA along with standard background therapy and the other group received only standard background therapy. The primary outcome measure in this study was the clinical assessment as per West Haven’s scoring at the end of one week of treatment. The secondary outcomes were to assess the fasting serum ammonia levels, liver function test (LFT) parameters, psychometric tests, and quality of life using the SF-36 questionnaire. Statistical analysis was performed using GraphPad Prism version 5 software (San Diego, CA: GraphPad Software).

Results: The baseline characteristics between both groups were similar and were non-significant (p>0.05) except for the West Haven criteria. The average age of the participants in the study was 50 years. The mean decrease in West Haven score at the end of one week of treatment in the group receiving LOLA was -1.52, whereas the other group receiving standard background therapy showed a decrease of -1.2 (p=0.0099). The mean decrease in fasting serum ammonia was -13.7 in the group that received LOLA and -11.7 in the other group, but this difference was not statistically significant (p=0.68).

Conclusion: In this study, we observed that administering LOLA in conjunction with standard background therapy has significantly improved the grade of HE, as determined by the West Haven scoring system. Fasting serum ammonia levels, LFT parameters, and quality of life as per SF-36 also improved in the group that received LOLA. Thus, the addition of LOLA to the first-line standard therapy was found to be effective and might help in improving the clinical grade of HE.

## Introduction

Hepatic encephalopathy (HE) is a reversible neuropsychiatric condition that occurs as a consequence of acute or chronic liver disease. It may arise spontaneously or develop as a result of some precipitating factor in the course of acute or chronic liver disease. HE was defined as "a spectrum of neuropsychiatric abnormalities in patients with hepatic insufficiency following the exclusion of other disorders of the central nervous system" [1].

HE is associated with severe cognitive impairment, and patients usually present with an acute confusional state with progressive disorientation, inappropriate behaviour, agitation, and finally progressing to coma [2]. Asterixis, extrapyramidal dysfunction, and hypertonia are some of the motor disorders observed in HE. There are various factors that can aggravate HE, which include renal failure, gastrointestinal bleeding (e.g., esophageal varices), constipation, infection, excessive dietary protein intake, dehydration, diuretics, diarrhea, vomiting, electrolyte imbalance, consumption of alcohol, etc. In some cases, creation of a transjugular intrahepatic portosystemic shunt (TIPS) can also lead to hepatic encephalopathy [3].

There are approximately 7-11 million cases of HE, with around 150,000 patients newly diagnosed each year. Approximately 20% of them present with cirrhosis [4]. The main factors implicated in hepatic encephalopathy pathogenesis are increased systemic and brain levels of ammonia. Ammonia enters the blood-brain barrier and starts to metabolize in astrocytes. The accumulation of glutamine in astrocytes, resulting from ammonia detoxification, leads to increased water entry and osmotic forces, ultimately inducing astrocytes to swell and causing cytotoxic edema [5].

Reducing the production of ammonia and maximizing the removal of ammonia from the bloodstream should be the main goals of HE treatment. The main agents used to treat overt HE are ammonia-lowering therapies, which include non-absorbable disaccharides, such as lactulose, and antibiotics such as rifaximin. Other therapies, such as oral branched-chain amino acids (BCAAs), intravenous L-ornithine L-aspartate (LOLA), probiotics, and other antibiotics, are also used per the American Association for the Study of Liver Diseases (AASLD) guidelines [2]. Lactulose, a type of non-absorbable disaccharide, is used for the treatment of overt HE. It decreases colonic transit time, which in turn reduces ammonia absorption into the circulation [6].

Rifaximin added to lactulose is the best documented agent to maintain remission in patients with one or more bouts of overt HE compared to lactulose treatment alone [7,8]. The BCAA causes detoxification of ammonia and corrects amino acid imbalance [9]. LOLA activates hepatic urea cycle activity and promotes glutamine synthesis, thereby decreasing ammonia levels. Oral and intravenous forms of LOLA have been shown to decrease serum ammonia levels in HE [10,11].

There is limited data available comparing LOLA with first-line treatments, such as rifaximin and lactulose. Studies related to the administration of LOLA in HE cases in the Indian setup are limited. Therefore, this study was designed to evaluate the additive effect of LOLA, along with rifaximin and lactulose, on the clinical outcomes in overt hepatic encephalopathy patients.

## Materials and methods

This study was conducted in the Department of Clinical Pharmacology and therapeutics in collaboration with the department of medical gastroenterology at a tertiary care hospital in Telangana. The study was conducted after obtaining ethical approval from the Ethics Committee of Nizam's Institute of Medical Sciences (70th ESGS No. 1554/2023), and it was registered with the Clinical Trials Registry of India (CTRI/2023/09/057449). The study was designed and conducted in accordance with the principles of Good Clinical Practice guidelines.

This is a prospective, observational, comparative, add-on, parallel-group study with a total duration of 12 months, from October 2023 to September 2024. Patients aged 18-65 years of either gender were included in the study. Patients with an overt stage of HE (grade 2 or more) as per West Haven criteria, admitted to medical gastroenterology wards, and only those who were able to comprehend letters and numbers were included in the study. Patients with serum creatinine >3 mg/dL and a history of intake of any drugs that may interfere with CNS activity were excluded from the study. Assuming a power of 80% and a level of significance of 5% (two-sided), for detecting a mean difference of 25.65 units and a pooled standard deviation of 30 units in fasting serum ammonia levels, the study required a total sample size of 48 [10]. Considering a dropout rate of 10%, the sample size was estimated to be 53. Considering unequal group sizes, 19 patients were included in group I (LOLA) and 34 in group II (standard).

The primary outcome measure in this study was the clinical assessment as per the West Haven scoring in both groups at the end of one week of treatment. The secondary outcomes were to assess the fasting serum ammonia levels, liver function test (LFT) parameters, psychometric tests, and quality of life using the SF-36 questionnaire at the end of one week of treatment. Adverse drug reaction (ADR) monitoring was done throughout the study.

Eligible subjects were enrolled from the department of medical gastroenterology wards. Written informed consent was obtained from patients who were conscious and coherent, or from their legally authorized representative (LAR) in cases where they were not. Re-consent was obtained from those patients who regained their consciousness at the end of the treatment period. Subjects diagnosed with hepatic encephalopathy (grade 2 or more) as per the West Haven scoring, who were on lactulose and rifaximin, were screened for eligibility. On the day of screening, demographic details, medical history, general and physical examination, vital parameters, lab investigations like complete blood count (CBC), liver function tests (LFT), renal function tests (RFT), chest X-ray, ECG, urine routine and examination (urine R/E) and details of background therapy were recorded in the case record form (CRF). All the participants who fulfilled the eligibility criteria were included in the study.

Eligible patients were followed up for one week. At the end of the week, those who received IV LOLA (20-30 g over 2-3 h) along with standard therapy for three to seven days were included in group I, while the remaining patients who received only standard therapy were included in group II. Standard background therapy included syrup lactulose 30 mL OD and tab rifaximin 550 mg BD given for three to seven days. Treatment was given at the discretion of the medical gastroenterologist. There were in total five visits with screening at day one (visit one), baseline visit at day 0 (visit two), during treatment visit at day three (visit three), end of treatment visit at day seven (visit four), and follow-up visit at day 14 (visit 5). Fasting serum ammonia levels were assessed on days 0 and seven. West Haven scoring was assessed on days 0, three, seven, and 14. Liver function test (LFT) and renal function test (RFT) values were assessed on days 0, three, and seven. Psychometric assessment using the number connection test-A (NCT-A) and number connection test-B (NCT-B) was done on day seven or at the end of treatment. Follow-up was done at day 14. Quality of life was assessed using the Short Form-36 (SF-36) questionnaire on days seven and 14. Patients were monitored very closely for any adverse events.

For clinical purposes, the West Haven criteria (WHC) are most frequently used for grading of HE [4]. The term "minimal" conveys that there are no clinical signs, cognitive or otherwise, of HE. The term "covert" includes minimal (grade 0) and grade 1 HE as per the West Haven criteria. Psychometric and neurophysiological testing strategies are used for the diagnosis [12,13]. Grade II and more are called overt HE as per the West Haven criteria.

The number connection tests can be easily done at the bedside and are the most frequently used psychometric tests. NCT-A test consists of connecting numbers in an arithmetic order (1-25) as quickly as possible. Time spent to complete the test is expressed in seconds, whereas the NCT-B test consists of linking numbers and letters in an alternative fashion (1-A, 2-B, 3-C), and the time taken is expressed in seconds [14].

Demographic data and clinical data at baseline were compared between the groups using an unpaired t-test, as the data followed a normal distribution. The mean change in the West Haven scoring, serum ammonia levels, and psychometric parameters at the end of treatment between the groups was compared using an unpaired t-test. The level of statistical significance was set at p<0.05. All statistical analyses were performed using the GraphPad Prism version 5 software (San Diego, CA: GraphPad Software).

## Results

**Figure 1:**
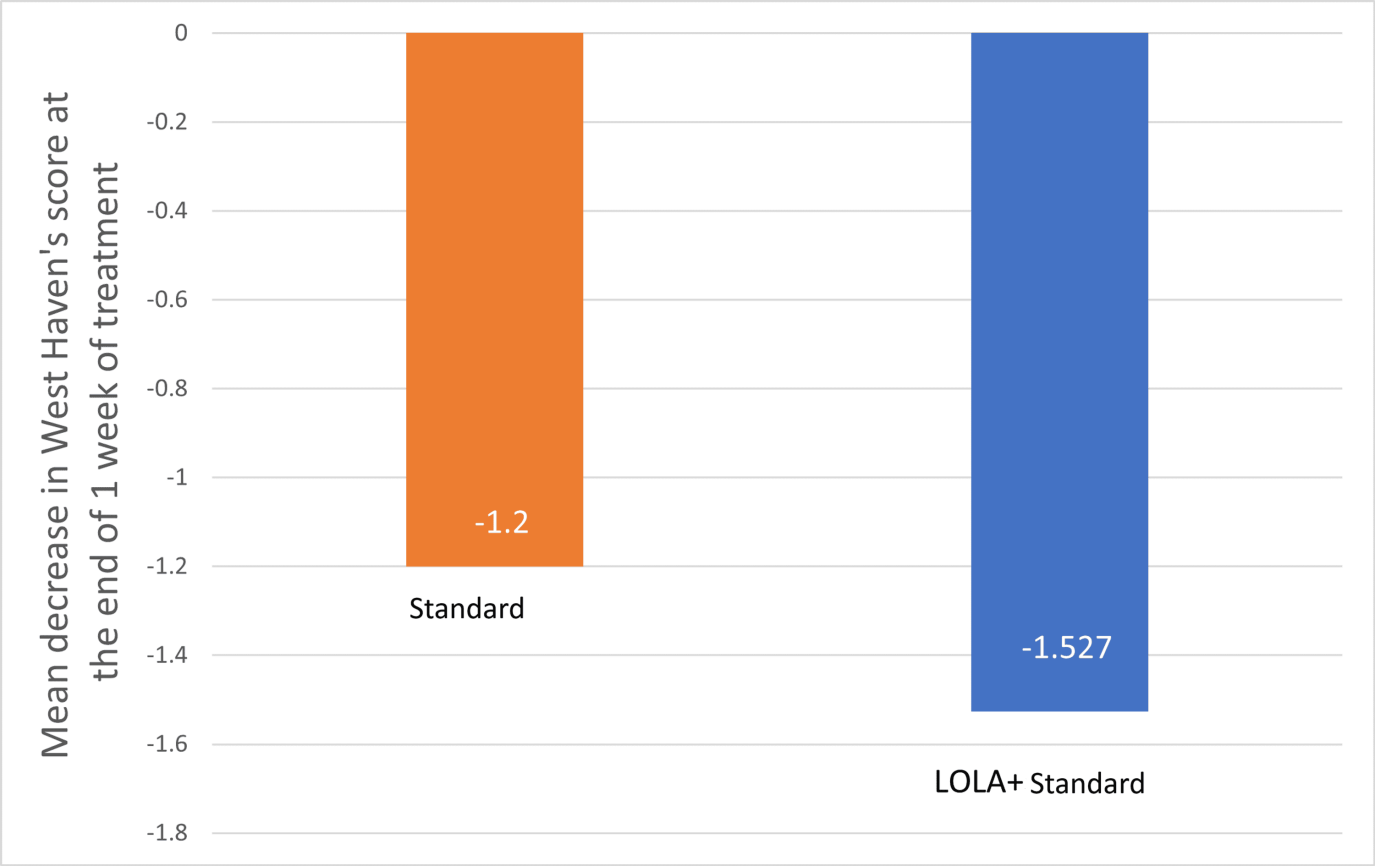
Mean decrease in West Haven scoring in the study groups at one week of treatment. LOLA: intravenous L-ornithine L-aspartate

A total of 61 patients were screened, and 53 were included in the study. Out of 61 patients, eight patients were screen failures because their serum creatinine levels were >3 mg/dL. There were 44 males and nine females. The mean age of the participants was 50 years. The number of diabetic patients was two in group I and four in group II, and the number of hypertensives was three in group I and four in group II. The baseline characteristics between both groups were similar and were found to be non-significant (p>0.05) except for the West Haven score. Baseline characteristics are presented in Table 1. The mean decrease in West Haven score at the end of one week of treatment in group I was -1.52 and group II was -1.2 (Figure 1). The result was found to be statistically significant (p=0.0099).

**Table 1:** Characteristics of study groups at baseline. LOLA: intravenous L-ornithine L-aspartate Values are presented as mean±SD. P<0.05 was statistically significant.

Characteristics	Group I (LOLA+standard) n=19	Group II (standard) n=34	p-Value
Age (years)	51.47±7.84	48.47±8.44	0.2
Gender (male:female)	15:04	29:05	0.62
				West Haven score	2.89±0.3	2.6±0.4	0.05
Fasting serum ammonia (mmol/L)	113.1±38.1	96.7±31.62	0.1
				AST (U/L)	100.8±35.2	94.17±43.14	0.56
ALT (U/L)	46.21±18.7	37.24±18.79	0.1				
				Serum albumin (g/dL)	3.07±2.063	2.50±1.01	0.18
Serum bilirubin (mg/dL)	5.268±4.08	4.94±4.74	0.8				

**Figure 2:**
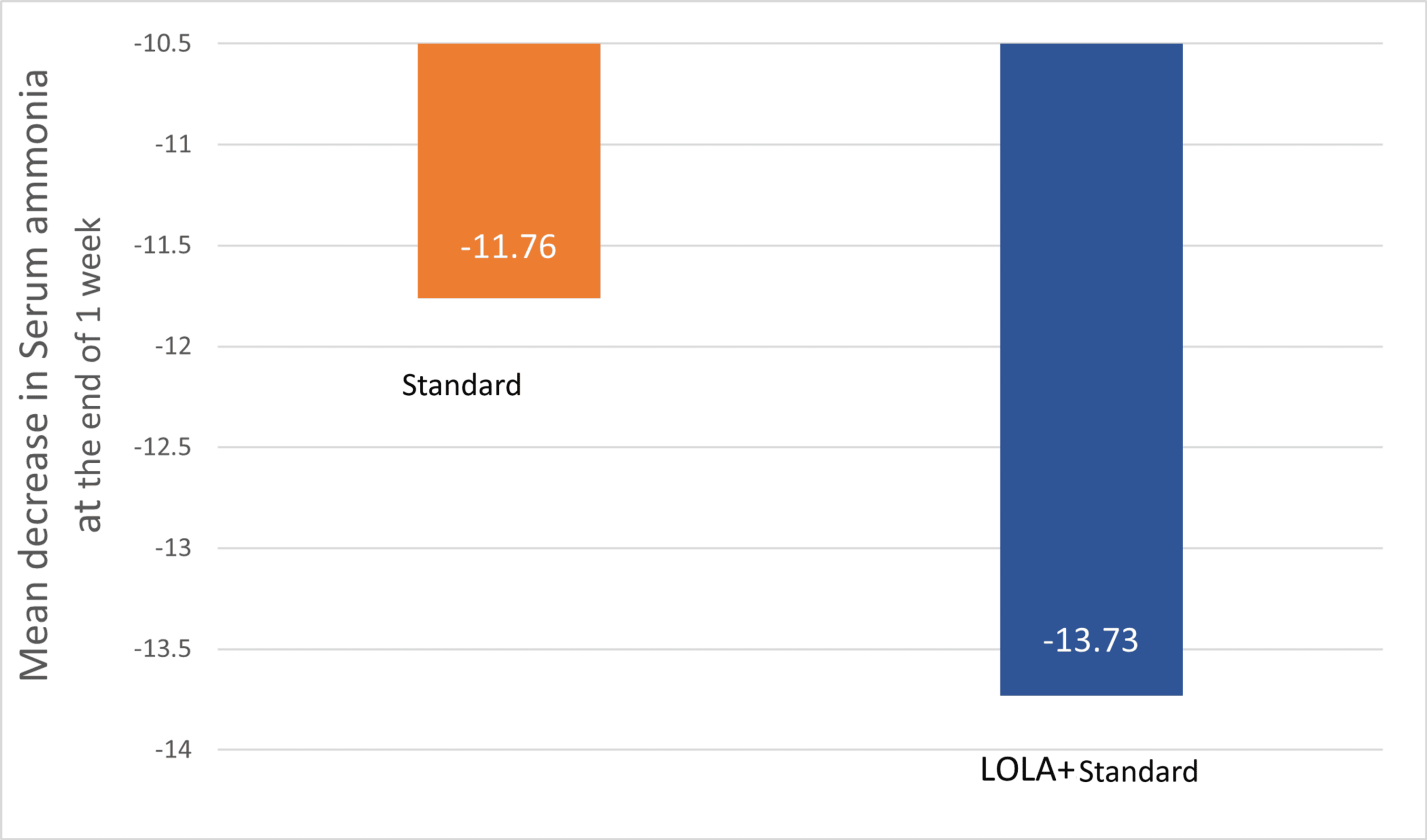
Mean decrease in fasting serum ammonia levels in the study groups at one week of treatment. LOLA: intravenous L-ornithine L-aspartate

The mean decrease in the fasting serum ammonia at the end of one week of treatment in group I and group II is shown in Figure 2. The mean decrease in group I (LOLA+standard) was by 13.7 units, and in group II, the mean decrease was by 11.7 units, but this was not statistically significant (p=0.68).

**Figure 3:**
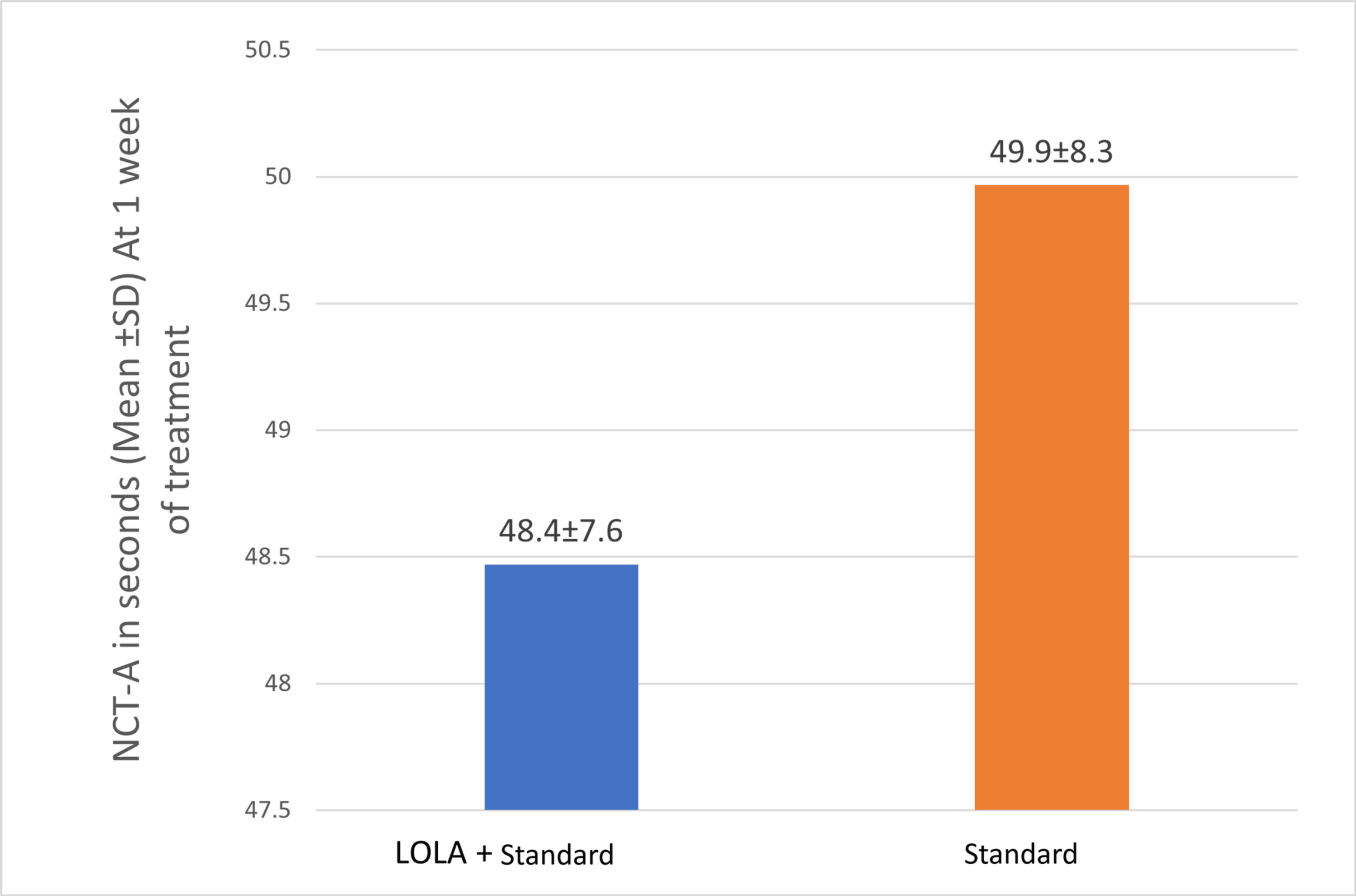
Mean NCT-A (in seconds) of the study groups at one week of treatment. LOLA: intravenous L-ornithine L-aspartate; NCT: number connection test

**Figure 4:**
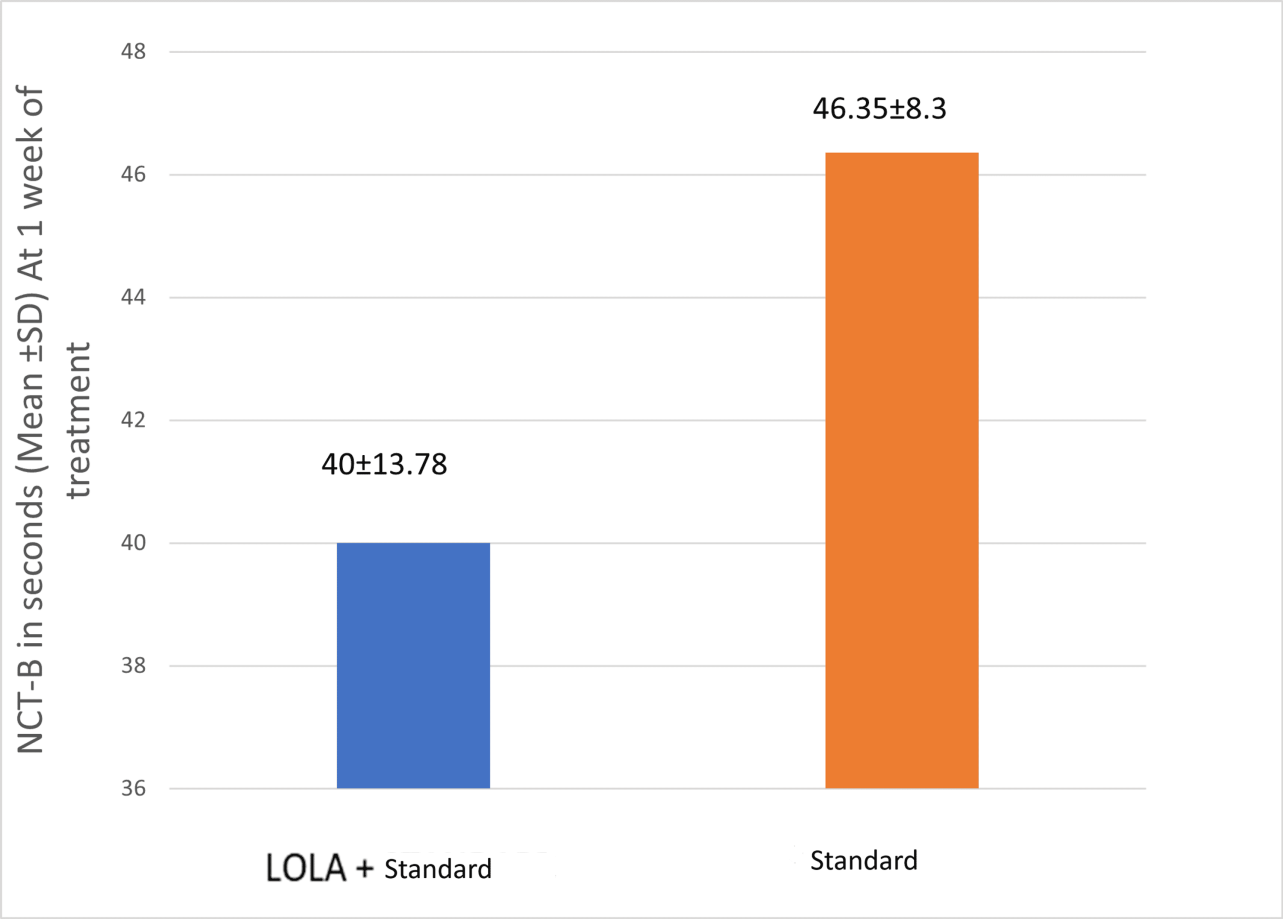
Mean NCT-B (in seconds) of the study groups at one week of treatment. LOLA: intravenous L-ornithine L-aspartate; NCT: number connection test

Psychometric tests, such as the number connection test-A and the number connection test-B, were used to compare clinical outcomes between the groups at the end of one week of treatment. It was calculated in seconds. The cut-off time for NCT-A and NCT-B is 1 min. Subjects whose cut-off time exceeded were excluded from the analysis. There was a decrease in the time taken to complete the NCT-A, NCT-B test in group I (LOLA+standard) compared to group II (standard) at the end of one week of treatment, but it was not statistically significant (p>0.05) (Figures 3, 4).

The LFT parameters (aspartate transaminase (AST), alanine transaminase (ALT), serum albumin, and serum bilirubin) were assessed at baseline and the end of treatment, one week later. There was a statistically significant decrease in their levels at the end of one week of treatment compared to baseline (p<0.05). When comparing the mean decrease in LFT parameters between group I and group II at the end of one week, statistical significance was only demonstrated for ALT (p=0.04). In contrast, for AST, serum albumin, and serum bilirubin, statistical significance was not demonstrated.

The SF-36 assessment scale was used to compare the quality of life (QoL) between the two groups at the end of treatment, on day seven, and at the end of the follow-up (F/U) visit on day 14 (Table 2). The QoL was better in group I compared to group II on day seven and day 14, but the difference was not statistically significant (p>0.05).

**Table 2:** Mean SF-36 score of the study groups on days seven and 14.

Groups	Day 7	Day 14	P-value between group I and group II (on day 7)	P-value between group I and group II (on day 14)
Group I (LOLA+standard)	56.63	66.79	0.368	0.9127
Group II (standard)	54.5	66.53		

There were no serious adverse drug reactions observed in both the study groups during the treatment and the follow-up period (Table 3). The most common adverse effects reported were nausea, headache, diarrhoea, and abdominal pain in both groups. All the ADR’s were mild, self-limiting, and resolved without any active intervention, and there was no requirement for prolonged hospitalization. None of the participants discontinued or were withdrawn from the study.

**Table 3:** Adverse drug reaction (ADR) events.

Adverse event	Group I (n=19)	Group II (n=34)
Nausea	1	0
Headache	1	0
Diarrhea and abdominal pain	0	1

## Discussion

Hepatic encephalopathy generally occurs in people with chronic liver disease, such as cirrhosis or hepatitis. Several triggering factors, such as infection and dehydration, may precipitate it. The treatment is mainly based on the removal of toxic substances from the intestine, which consists of administering drugs to reduce the production of ammonia.

Lactulose remains the mainstay of treatment. Lactulose has shown its efficacy in different clinical trials [15,16]. In recent times, rifaximin has been demonstrated to be as good as lactulose but has an increased chance of bacterial resistance. An increasing body of evidence in recent times has suggested that LOLA, a stable salt of natural L-amino acids, including ornithine and aspartic acid, exhibits direct hepatoprotective properties in chronic liver disease and has also been found to decrease the production of ammonia.

In this study, most patients (38 out of 53) had grade 3 HE as per West Haven scoring at baseline. There was a statistically significant decrease in the score in both groups at the end of treatment, one week after initiation (p<0.05). The mean decrease in West Haven score was greater in group I (LOLA+standard) compared to group 2 (standard), and this difference was statistically significant (p=0.0099). This was consistent with previous studies. A study done by Sidhu et al. showed that in patients with bouts of overt HE, IV LOLA as an add-on therapy to lactulose and rifaximin has significantly improved the grade [17]. In another placebo-controlled trial conducted by Jain et al., it was found that there was an improvement in the grade of HE in the LOLA group compared to placebo [18].

One of the secondary outcome measures in this study was the assessment of fasting serum ammonia. There was a significant reduction in serum ammonia in both groups at one week of treatment (p<0.05). The mean reduction of serum ammonia in group I (LOLA+standard) was more pronounced by -13.73 compared to group II (standard), which was -11.76; however, this difference was not statistically significant (p=0.68). Poo et al. conducted a study in 2006, which found that both the lactulose group and the LOLA group experienced a significant decrease in ammonia levels (p<0.05) [19].

In a study done by Sidhu et al., it was observed that in patients with overt HE, intravenous LOLA, when added to lactulose, significantly improved the grade of overt hepatic encephalopathy (OHE) and decreased the level of venous ammonia [17]. In another trial, patients with cirrhosis were randomized to receive LOLA or a placebo. Patients were assessed using the critical flicker frequency test and arterial ammonia levels. At follow-up, the change in the ammonia level was significant in patients treated with LOLA compared with placebo [20].

The other secondary outcomes in this study were the psychometric tests NCT-A and NCT-B, measured at one week of treatment. The mean NCT-A (in seconds) of group I (LOLA+standard) was 48.47±7.6, which was lower than group II (standard), which was 49.9±8.3, but it was not statistically significant (p=0.544). Poo et al. in their study also found that patients receiving LOLA had a significant improvement in their NCT-A scores compared to the lactulose group (184±43 versus 88±7) [19]. In this study, there was a decrease in the time taken to complete the NCT-B test in group 1 (LOLA+standard) compared to group II (standard), although the difference was not statistically significant (p=0.06). However, none of the other studies reported the effect of LOLA on other psychometric tests, such as the NCT-B, in HE patients.

LFT parameters (ALT, AST, serum albumin, serum bilirubin) were also used as secondary outcomes in this study. A statistically significant decrease in ALT levels was observed at the end of one week of treatment within and between the groups (p<0.05). There was also a decrease in AST and serum bilirubin levels, and an increase in serum albumin levels within the groups compared to baseline, which was statistically significant (p<0.05). However, between-group comparisons did not show any statistically significant change in their levels. These results are consistent with previous studies. Najmi et al. in their study found that the change in ALT levels was significantly different between the LOLA (-126±64.2) and the control group (19.3±128.2) [20,21].

On doing QoL assessment using the SF-36 assessment scale, it was found that the QoL was better in group I compared to group II on days seven and 14; however, this difference was not statistically significant (p>0.05). Poo et al. in their study observed that there was improvement in SF-36 QoL in the LOLA group (47±22 to 54±21) compared to the lactulose group, where it decreased (42±19.5 to 41±15.3) [20]. The study drugs were well tolerated. Mild adverse effects like nausea, headache, diarrhea, and abdominal pain were noted. They were self-limiting.

This study was done to assess the role of LOLA in the treatment of patients with overt hepatic encephalopathy. Most studies published in the literature have assessed the effect of LOLA only on the NCT-A test, but in this study, we have tried to evaluate the effect of LOLA on other additional psychometric parameters, like the NCT-B.

Study limitation

In this study, we only evaluated the effect of LOLA on overt HE patients. The assessment of SF-36 at baseline was not conducted in this study, as patients were in the overt stage of HE, which may have prevented them from filling out the questionnaire-based form.

## Conclusions

The present study compared the additive effect of LOLA with standard background therapy, rifaximin, and lactulose. In this study, we observed that administering LOLA in conjunction with rifaximin and lactulose significantly improved the grade of HE, as determined by the West Haven scoring, compared to the group that received only lactulose and rifaximin, and this difference was statistically significant. Fasting serum ammonia levels were also decreased in the group receiving LOLA.

A trend of improvement was seen in the LFT parameters (AST, ALT, serum albumin, serum bilirubin), and quality of life as per SF-36 also improved in the group that received LOLA. Thus, the addition of LOLA to the first-line standard therapy was found to be effective and might help in improving the clinical grade of HE. However, further long-term studies with a larger sample size and at different stages of HE are required to explore and correlate the beneficial effects of LOLA in this direction.
